# How Do COVID-19 Vaccine Policies Affect the Young Working Class in the Philippines?

**DOI:** 10.3390/ijerph20032593

**Published:** 2023-01-31

**Authors:** Rey Hikaru Y. Estoce, Olivia M. Y. Ngan, Pacifico Eric E. Calderon

**Affiliations:** 1 St. Luke’s Medical Center College of Medicine—William H. Quasha Memorial, Quezon City 1112, Philippines; 2 Medical Ethics and Humanities Unit, School of Clinical Medicine, LKS Faculty of Medicine, The University of Hong Kong, Hong Kong SAR, China; 3 Centre for Medical Ethics and Law, Faculty of Law and LKS Faculty of Medicine, The University of Hong Kong, Hong Kong SAR, China; 4 Clinical Ethics Services, St. Luke’s Medical Center, St. Luke’s Medical Center—Quezon City and Global City, Metro Manila 1634, Philippines

**Keywords:** vaccine, vaccine policy, public health, inequality, Philippines

## Abstract

Dubbed the “inequality virus”, coronavirus disease (COVID-19) has unveiled and magnified many of the global society’s long-standing inequalities and health inequities. This work brings together the phenomena of increased inequality and health inequities felt by the poor and young working class of the Philippines and how they interact negatively with existing vaccine policies. The poor and the young were more likely to have experienced employment disruptions with limited access to technologies that allowed for teleworking. Informal economy workers suffered from diminished labor protection and draconian lockdowns. Disadvantaged areas persistently dealt with limited health resources, and the working class was disproportionately vulnerable to COVID-19 infection. Utilitarian vaccine policies such as mandatory vaccination and the prioritization scheme negatively interacted with these COVID-induced inequalities and health inequities. While the young working class was more likely to be unemployed, mandatory vaccine policy required that they get vaccinated before seeking re-employment. However, the prioritization scheme adopted by the government failed to target them as a priority. This left them in a vulnerable state of prolonged unemployment while on standby for better supply and improved infrastructure for vaccine rollout. Future prospects in terms of economic recovery and health equity will be affected by issues such as potential increased taxation, the rapidly digitalizing labor market that is evolving to favor highly-skilled workers, and the staging of universal healthcare in the country.

## 1. Background

The COVID-19 pandemic resulted in unprecedented disruption to economies and 31 labor markets globally, affecting various sectors of society in distinct ways during the national lockdown. The shift from onsite to remote working options in securing operational continuity was feasible for industries amenable to digital adoption, which are likely the highest income-earning occupations [1]. However, some low-income sectors, such as hospitality, retail, and food and beverage services, encountered financial hardship and were closed due to operational barriers where remote work is infeasible. Individuals working in these sectors were mostly ‘informal’ workers from low-income households, facing critical job losses, compared to others working in a secure market [2]. Labor analysis showed that the global recession posed an immediate financial impact among individuals employed in these shutdown sectors, who were more likely to be the youth than their older counterparts. For instance, individuals younger than 25 years in the UK were found to be 2.5 times more likely to work in industries that were shut down than other age groups [1]. In Slovenia, around three times more younger people aged 18 to 29 years found themselves along an unstable financial trajectory compared to the others [3]. Intergenerational disparity worsens due to disruption to economies and labor markets, posing a disproportionate effect on the young population. Young individuals are at a lower risk of developing severe physical symptoms than older cohorts. They, however, are more vulnerable to securing quality jobs and income in the economic decline because the pandemic has disrupted their access to education and employment opportunities. Income disparity by age will become entrenched when an economic recession becomes a long-term problem.

This paper aims to (1) discuss the ethical considerations of two major vaccine policies, (2) describe the interaction of vaccine policies and the difficult situation faced by young and informal workers, and (3) offer a critique of such policies in the context of COVID-19 inequity.

## 2. Philippine Context

### 2.1. Employment Disruption: High Unemployment among the Young Informal Workers

The high transmissibility of COVID-19 and government responses related to closure and containment lead to unprecedented challenges to local health systems, economic consequences, and epidemiological progression [4]. In the Philippines, the country recorded more than 4.0 million cases and 65,000 deaths at the time of the writing, noting one of the highest mortality rates in the Southeast Asia Region. The government imposed stringent public health measures since the COVID-19 outbreak, including travel restrictions, community quarantine, and active testing. However, the system struggled to flatten the curve due to limited capacity and resources in the initial phase [5]. Table 1 describes the major key events in the COVID-19 outbreak.

In response to the pandemic, the Philippine government passed a law called Bayanihan to Heal as One Act, a particular emergency policy granting the President powers necessary to carry out urgent measures to meet the current national emergency related to COVID-19 [6]. The scope broadly includes but is not limited to penalizing individuals or groups violating measures, providing an emergency subsidy to low-income households, and providing public health workers with an allowance. The unrestricted scope of the policy raised heavy public criticism for giving law enforcers too much discretion.

Through time, although the number of COVID-19 cases declined, the income per working member failed to recover to pre-pandemic levels as of October 2020 [7]. A World Bank survey reported that about 40% of low-income households had no earnings reported during one of the most prolonged lockdowns enforced throughout the country [7]. Two out of five individuals working in the low-wage “non-essential” precarious forces supporting communities’ operations were minimally protected and vulnerable to economic disruptions [8]. Among the socioeconomic factors, the youth is likely the population engaging in “non-essential” work in the middle of an unemployment crisis. At the onset of the pandemic, the unemployment rate among individuals aged 15 to 24 years was 13.6%, which was triple-folded or sextuple-folded higher compared with other groups aged 25–54 and 55 and older, respectively. In June 2022, the youth unemployment rate remained at 14.5%, around double that of the general population at 7.7% [9]. While it is plausible that a structural issue exists, our intention is to highlight how the increase in youth unemployment was disproportionately higher when compared to their older counterparts during the pandemic, signaling the negative effects of the pandemic and public health policies on the youth. In the middle of the unstable employment and wage landscapes presented above, we aim to discuss how the younger working class was implicated by the pandemic and how COVID-19 vaccination was perceived to alleviate this problem.

### 2.2. Two Major Vaccine Policies: Neglecting Young Informal Workers

Mass vaccination is considered one of the most effective programs for protecting individuals against COVID-19. There are two major ethical considerations related to vaccination around (1) allocation and (2) mandate. The following sections discuss the related issues in the Philippines context.

#### 2.2.1. Prioritization Framework

The Philippines is one of the beneficiaries of the COVAX program, a global initiative co-led by the Coalition for Epidemic Preparedness Innovations, Gavi, WHO, and UNICEF to promote vaccine equity. As of May 2022, the Philippines received 245 million COVID-19 vaccines in several batches, which was insufficient to cover primary and booster doses for the entire national population of 115 million [10]. The limited supply requires careful consideration of equitable allocation.

The Vaccination Prioritization Framework was implemented to allow the two doses based on public health goals of reducing the mortality rate and preserving the health system capacity [11]. The aim was to prioritize limited vaccine doses in a fair and equitable manner. The first dose was administered to the general population without selection or exclusion. The second booster was prioritized for vulnerable groups based on the risk of exposure in three groups. The prioritized group was primarily those at a high risk of death or severe symptoms and increased risk of infection, including populations at the highest risk of disease and death, such as healthcare workers, personnel in essential sectors, senior citizens, individuals with comorbidities, and impoverished people. The next group included individuals living or working at a lower risk of infection, such as teachers, social workers, government workers, essential workers, overseas Filipino workers, and socio-demographic groups at significantly higher risk than senior citizens, poor populations, and other workforces. The last group was another population not otherwise included in the above groups, such as the young and informal workers.

**Figure 1:**
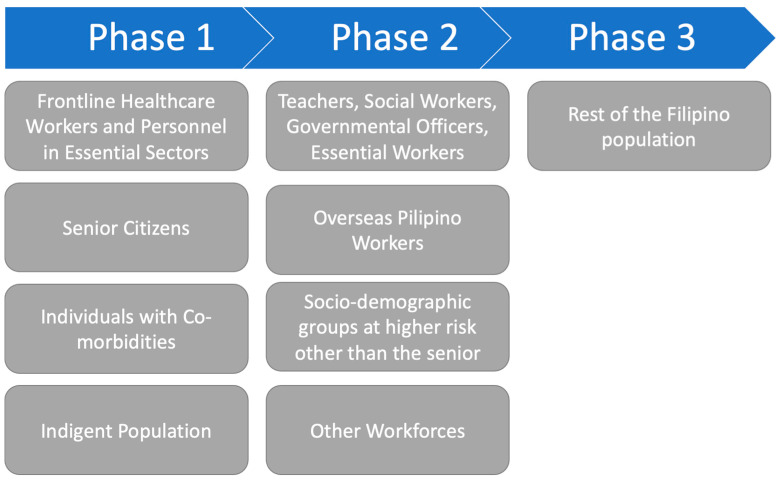
Vaccine Prioritization Framework [13].

The vaccination prioritization framework (Figure 1) manifested as an implicit utilitarian aspiration of the prioritarian theory of justice, giving priority to the worse-off [11,12]. This view implies that scarce resources should be allotted to maximize the benefits of the number of lives saved and prioritize at-risk populations to prevent morbidity and mortality.

#### 2.2.2. Mandatory Vaccine Policy

Globally, the government mandates vaccination to exercise the duty to care necessary for and proportionate to the achievement of societal and public health goals in protecting the population’s well-being. The Philippines’ public and private sectors also require employees to provide evidence of their COVID-19 vaccination status to continue their work. Others who remain unvaccinated or receive an authorized exemption to vaccination are required to undergo RT-PCR tests regularly at their own expense [14]. Both work and the mandatory vaccine policy affect daily life. The Department of Transportation imposed a “no vaccination, no ride” policy, banning unvaccinated commuters from riding public transport within Metropolitan Manila [15]. The mandatory “no vaccine, no work” policy did not come into effect without dispute. Some emphasized that it is unjust for those unvaccinated to regularly be tested for COVID-19 at their own expense when wages remain insufficient for the most general population in consideration of economic challenges. Several civic organizations petitioned the Supreme Court to nullify the “unconstitutional and violative” policy while situating it in the context of immense corruption in health care, mass media, scientific reporting, and elections [16].

A major ground justifying mandatory vaccine policy enforcement is based on herd immunity, which can be interpreted as a public good from which many people derive a non-excludable benefit and is a prime goal for the utilitarian [17]. Giubilini et al. argued that this act might be interpreted as a contribution, albeit seemingly negligible, to attaining a collective effect wielded towards achieving the best outcome in which people benefit the most [18]. Similarly, Pierik’s assertion that mandatory vaccination should be enforced by governments to “guard the common good of herd immunity to protect vulnerable persons” resorts to Utilitarianism [19].

## 3. COVID-19 Vaccine Policies and Inequity: The Unemployed and Unprioritized

Vaccination for informal sector workers commenced only fifteen months after Duterte declared a public health emergency [20]. This was when vaccine coverage in the Philippines was 6.6% while neighboring countries such as Singapore had initially vaccinated half of their population, and Cambodia (20%), Malaysia (14%), and Brunei (12%) reported higher vaccination rates [21]. Meanwhile, the labor force participation rate was 65%. The unemployment rate was 7.7% [22], higher than the 5.1% pre-pandemic rate in 2019 [23].

Vulnerability among the poor and working class highlighted the widening gaps in health inequities. In 2019, the Universal Healthcare (UHC) Act was enacted to expand the National Health Insurance Program, improve access to quality healthcare, and attain health-related Millennial Development Goals. The landmark legislation also aimed to alleviate the “triple burden” of non-communicable diseases, communicable diseases, and the effects of globalization and climate change [24]. However, despite efforts to stage UHC, the country’s health system remains fragmented. This is mirrored in inequity bared when a disaggregated analysis is made regarding resource allocation in the archipelago. This means that geographically isolated and disadvantaged areas, where poverty remains rampant, make do with scarce resources, while highly urbanized areas, such as the National Capital Region, enjoy a cornucopia [25]. Vaccine rollout was also marred with issues of VIP vaccination, with the elite skipping lines to receive the highly sought-after early allocations, most evident in areas elusive to “imperial Manila” [26].

## 4. Outlook: Sink or Swim?

Moving forward has set out a repertoire of both potential challenges and opportunities. Response to the ongoing pandemic and its economic ravages bears crucial implications for socioeconomic and health inequities for the Filipino people. Among the defining events discussed here are taxation, the changing labor market, and UHC.

After the May 2022 national polls, where President Ferdinand Marcos Jr. garnered a historic majority vote, many are keen to see how his administration’s economic team will fare in the wake of Duterte’s term, which has left a massive P12.68 trillion outstanding debt. During the transition to the new administration, outgoing National Economic Development Authority chief Karl Chua urged the Marcos administration to continue its policy agenda to boost economic recovery [27]. One of the finance department’s proposals included postponing a scheduled tax cut for those earning P250,000 to P400,000 per annum. Initially, the personal income tax rate should be reduced from 20 to 15 percent from 2023 onwards, as stipulated in the Tax Reform for Acceleration and Inclusion Law. It is projected that deferring this for three years will accumulate an estimated P97.7 billion per year, which will help pay off the national debt [28]. These fiscal policies will undoubtedly concern the working class, who are to pick up the pieces after the economic onslaught of the pandemic while potentially being taxed heavily. Whether the current administration will adopt these proposals is yet to be cleared. Incoming finance secretary Benjamin Diokno has expressed non-favor, suggesting that other means, such as optimizing revenue collection and wise budget allocation, as more viable options [29].

COVID-19′s lasting impact is felt in many spheres, including the labor market. It has expedited digitalization, which has either transformative or destructive effects depending on the sector [8]. Workers are increasingly forced to learn and reinvent as industries innovate and adapt to telecommuting as a norm. It will be an added burden for those without access to home computers and a stable internet connection. Not to mention, these are usually highly-skilled jobs that will more likely be afforded to the educated. In fact, the massive reallocation of jobs was towards those in sectors such as communication, technology, and other higher-skilled services [30]. For instance, there has been a spike in need for cybersecurity expertise due to increasing threats in digitalization. Regardless, the country fails to produce those skillful enough to fill this demand due to a lack of institutionalized courses [31]. In response, the education sector and government have the opportunity to capacitate and train workers in these fields while demand is high, such as by offering free or subsidized trade courses responsive to the industry’s needs. The evolution of the labor force to be in sync with the changing labor market is another phenomenon that will shape the covidized future of the “lockdown generation”.

In terms of health prospects, the pandemic has put up vital challenges in the health system that need refinement. UN Secretary-General Antonio Guterres poetically put it, “COVID-19 has been likened to an x-ray, revealing fractures in the fragile skeleton of the societies we have built. It exposes fallacies and falsehoods everywhere: The lie that free markets can deliver healthcare for all…” [32]. Challenges for the country during the early phases of the pandemic highlight the health system’s lack of preparedness to respond to public health emergencies. The inequitable distribution of both human and health resources in the devolved system was indicated by Amit et al. as the primary reason for this [5]. While the UHC Act’s goal has been to address these very problems, it has taken a back seat as the country grappled with containment measures. As we foray into the “New Normal for Health,” the Department of Health has set out strategies to catch up on the losses for UHC [33]. Salient among them are the utilization of telemedicine and telehealth to improve health service delivery, an improvement in the capacity to handle and respond to health emergencies, streamlining digitalized processes for epidemiologic surveillance, maximization of multi-sectoral engagement, and health promotion. Years after its passage into law, UHC is yet to be fully implemented and felt, and its effects on reducing inequities are yet to be seen. For the young working class, these promises will undoubtedly play out importantly as members of this class hope to rebuild their lives with a just health system they can depend on.

## 5. Conclusions

One of the many ill effects of the COVID-19 pandemic is its exacerbation and interaction with existing health inequities and inequalities. The young working class of the Philippines, especially those in the informal sector, face magnified challenges such as increased employment disruption and a greater risk of developing COVID-19. Utilitarian vaccine policies such as mandatory vaccination and the vaccine prioritization scheme put the young working class in a position of prolonged unemployment and being behind regarding receiving the vaccine. Prospects for this group include potential increased taxation, a rapidly digitalizing labor market, and the laying out of universal healthcare. Policy response will play a defining role as the country gears towards a post-COVID-19 world defined by volatility and increased globalization.

## Figures and Tables

**Table 1:** Key events of COVID-19 pandemic in the Philippines.

Timeline	Event(s)
30 January 2020	Index Patient arrives in the Philippines A 30-year-old female Chinese national who visited the country for leisure
7 March 2020	The first local transmission of COVID-19 was confirmed
9 March 2020	President Rodrigo Duterte issued Proclamation No. 922, declaring the country under a state of public health emergency
12 March 2020	Metro Manila was placed under partial lockdown to prevent a nationwide spread of the virus
16 March 2020	The entire Luzon was put under “enhanced community quarantine”
17 March 2020	State of Calamity throughout the Philippines and an imposed Enhanced Community Quarantine throughout Luzon
25 March 2020	Bayanihan Heal as One Act was enacted

## Data Availability

Not applicable.
